# Evaluation of the Osteogenic Potential of Different Scaffolds Embedded with Human Stem Cells Originated from Schneiderian Membrane: An* In Vitro* Study

**DOI:** 10.1155/2019/2868673

**Published:** 2019-01-15

**Authors:** Rita Bou Assaf, Mohammad Fayyad-Kazan, Fatima Al-Nemer, Rawan Makki, Hussein Fayyad-Kazan, Bassam Badran, Antoine Berbéri

**Affiliations:** ^1^Department of Oral and Maxillofacial Surgery, Faculty of Dentistry, Lebanese University, Beirut, Lebanon; ^2^Laboratory of Cancer Biology and Molecular Immunology, Faculty of Sciences-I, Lebanese University, Hadath- Beirut, Lebanon

## Abstract

**Background:**

Novel treatments for bone defects, particularly in patients with poor regenerative capacity, are based on bone tissue engineering strategies which include mesenchymal stem cells (MSCs), bioactive factors, and convenient scaffold supports.

**Objective:**

In this study, we aimed at comparing the potential for different scaffolds to induce osteogenic differentiation of human maxillary Schneiderian sinus membrane- (hMSSM-) derived cells*. Methods*. hMSSM-derived cells were seeded on gelatin, collagen, or Hydroxyapatite *β*-Tricalcium phosphate-Fibrin (Ha*β*-TCP-Fibrin) scaffolds. Cell viability was determined using an MTT assay. Alizarin red staining method, Alkaline phosphatase (ALP) activity assay, and quantitative real-time PCR analysis were performed to assess hMSSM-derived cells osteogenic differentiation.

**Results:**

Cell viability, calcium deposition, ALP activity, and osteoblastic markers transcription levels were most striking in gelatin scaffold-embedded hMSSM-derived cells.

**Conclusion:**

Our findings suggest a promising potential for gelatin-hMSSM-derived cell construct for treating bone defects.

## 1. Introduction

In clinical procedures, the use of bone grafts from distinct origins (autograft, allograft, or xenograft) is a standard approach for treating bone defects caused by traumas, bone tumors, or birth flaws. Although autografts are commonly used, many complications can arise including pain, infection, scarring, and donor-site morbidity [1]. On the other hand, allografts do not perform as well as autografts due to their lower osteoactive potential, as well as the high risk of immune rejection and infectious pathogens transferring [2, 3]. Such limitations encouraged the search for clinically relevant engineered structures, designated as bone graft substitutes, which constitute a combination of artificial extracellular matrix (ECM) scaffold, bone progenitor cells that can differentiate into osteoblasts, and/or growth factors [4]. This combination has proven significantly efficacious in animals and humans [5–9]. Scaffolds, made of distinct natural and/or synthetic components, provide a 3-dimensional environment that closely mimics the native tissue [10, 11]. Nowadays, a variety of scaffolds such as collagen, gelatin, chitosan, hydroxyapatite HA, and tricalcium phosphate (TCP) are being used [12, 13]. Besides being nontoxic and nonimmunogenic, the structure and chemical composition of the scaffold should support cell adhesion, viability, proliferation, homing, and differentiation [10, 11]. Following implantation to the injured site, the diffusing interstitial fluid will be the only source of nourishment for the cells on the biomaterial [14, 15]. Biological activities are,* therefore*, directly affected by the material characteristics, including biocompatibility and surface chemistry, that can alter the responsiveness of specific cells. Mesenchymal stem cells (MSCs) derived from distinct tissues including bone marrow, adipose tissue, and dental and periodontal tissues are the most commonly used sources to generate osteoprogenitor cells [16–23]. Human maxillary Schneiderian sinus membrane- (hMSSM-) derived cells are mesenchymal stem cells (MSCs) characterized by a spindle-shaped fibroblast-like morphology and a high expression of mesenchymal makers, such as STRO-1, CD44, CD90, CD105, and CD73 [24]. Histologically, hMSSM is made up of different layers including a ciliated columnar epithelial lining on the internal side and a highly vascularized lamina propria and periosteum on the osseous side [25]. The periosteum of the maxillary bone contains osteoprogenitor cells that can be isolated and expanded in culture and transplanted,* in vivo*, to trigger ectopic bone formation [26]. Recently, researchers have demonstrated that maxillary sinus membrane lifting, without insertion of any grafting material, is a suitable technique for augmenting the volume of the maxillary sinus bone floor. However, there has been some controversy over how to maintain the sinus membrane elevated, and what material to place inside the sinus cavity [27–32]. Scaffolds provide a space maintainer for the sinus membrane, can serve as a matrix that supports the proliferation and migration of local osteoprogenitor cells, and subsequently trigger bone formation [33–35]. Interestingly, several* in vitro* and* in vivo* studies demonstrated that hMSSM-derived cells are capable of differentiating into cells of osteogenic lineage, thus holding a great clinical promise for better implant-based therapies [24, 25, 36, 37]. Aware that collagen, gelatin, and hydroxyapatite/tricalcium phosphate/Fibrin (HA/*β*TCP/Fibrin) scaffolds are characterized by many advantageous features including nonimmunogenicity, biocompatibility, and bioactivity and commonly applied in dental and craniofacial regeneration [38–41], we evaluated in this* in vitro* study the ability of these scaffolds to induce osteogenic differentiation of hMMS-derived cells. This* in vitro* study could, therefore, simulate the first step of bone regeneration following sinus lift.

## 2. Materials and Methods

### 2.1. Scaffold Preparation

#### 2.1.1. Hydroxyapatite and Tricalcium Phosphate (HA/*β*TCP) Scaffold

Sterile hydroxyapatite/tricalcium phosphate (HA/*β*TCP) granules (Osteon III™, GENOSS Co., South Korea) comprising 60% hydroxyapatite (HA) + 40%  *β*-Tricalcium Phosphate (*β*TCP) of 0.25-1 mm particle size were used in this study. Confluent cultures (P3) were trypsinized and then washed with PBS. 3 × 10^6^ cells were mixed with 40 mg of HA/*β*TCP granules in separate tubes and rotated gently in the incubator (37°C/5% CO2). After two hours, cells with attached particles were collected by brief centrifugation and then mixed with human fibrinogen (5 mg/ml) and thrombin (5U/ml in 25 mM CaCl2). After polymerization into Fibrin HA-*β*TCP biphasic granulate at 37°C, additional media were added. Another group of HA- *β*TCP scaffolds without cells were mixed with Fibrin gel, incubated with culture medium alone, and used as a control.

#### 2.1.2. Hemostatic Gelatin Sponge Scaffold

Sterile hemostatic gelatin sponges (10 × 10 × 5 mm cubes) (Cutanplast®* Mascia Brunelli S.p.a*.* V. le Monza, Milano, Italy*) were soaked in *α*-MEM (Sigma-Aldrich, USA) containing 10% FBS, 1% PS, and 2Mm L-glutamine (nonosteogenic media) for 1 hour and then placed into 48-well plates. A drop of 50 *μ*L containing 3 × 10^6^ cells was placed on top of each scaffold and allowed to attach for 2 hours in the incubator (37°C, 5% CO2). After two hours, additional media were then added on the top of gelatin scaffolds. Cell-free scaffolds incubated with culture medium alone were used as control.

#### 2.1.3. Collagen Scaffold

A sterile collagen wound dressing tape (CollaTape, Zimmer Biomet Dental, Palm Beach Gardens, FL, USA) was cut (10 × 10 mm), soaked in *α*-MEM containing 10% FBS, 1% PS, and 2Mm L-glutamine (nonosteogenic media) for 1 hour, and then placed into 48-well plates. A drop of 50 *μ*L containing 3 × 10^6^ cells was placed on top of each scaffold and allowed to attach for 2 hours in the incubator (37°C, 5% CO2). Two hours later, additional media were then added on top of collagen scaffolds. Cell-free scaffolds incubated with culture medium alone were used as control.

### 2.2. HMSSM-Derived Cells Isolation, Cultivation, Characterization, and Osteogenic Differentiation

This study was approved by the Ethical Committee of the Lebanese University. Samples were obtained according to ethical guidelines, after informed consent forms were signed by the patients enrolled in the study. We followed the same procedure of Berberi et al. 2016 [24]. A total of 18 human maxillary Schneiderian sinus membrane (hMSSM) samples (~2 × 2 cm) were obtained during a surgical nasal approach for treatment of chronic rhinosinusitis, performed under general anesthesia. Smokers and patients with skeletal disorders or systemic diseases were excluded from the study. After collection, tissue samples were placed in phosphate buffered saline (PBS) containing 1% penicillin-streptomycin (P/S) at 4°C and processed within 24 hours, as described in our previous study [24].

#### 2.2.1. Isolation of hMSSM-Derived Cells

We followed the method described by Berberi et al. 2016 [24]. hMSSM samples were extensively washed with PBS supplemented with 1% P/S and cut into small pieces under aseptic conditions. Tissue fragments were incubated with 1 U/ml dispase I solution (Sigma-Aldrich, USA) in PBS at 37°C for 1 hour to separate the epithelial lining from the membrane. Epithelial cells were discarded, and the remaining tissue fragments were treated with 200 collagen digestion units (CDU)/ml of collagenase type II (Sigma-Aldrich, USA) in Hank's balanced salt solution (HBSS) containing 5 Mm calcium chloride at 37°C for 3 hours. The tissues were shaken repeatedly during enzymatic incubation. The resulting cells were filtered out with a 40 *μ*m cell strainer (BD Bioscience). The hMSSM-derived cells were then centrifuged at 900 RPM for 10 minutes.

#### 2.2.2. Culture of hMSSM-Derived Cells in Nonosteogenic Conditions

We followed the procedure of Berbéri et al. 2016 [24]. Briefly, isolated cells were plated in T75 cm^2^ with alpha-minimum essential medium (*α*-MEM) (Sigma-Aldrich, USA) containing 10% fetal bovine serum (FBS), 1% penicillin-streptomycin (PS), and 2Mm L-glutamine (nonosteogenic media) and cultured in an incubator at 37°C, 5% CO_2_. Daily morphologic characterization was done using an inverted microscope, and the culture solution was changed twice a week. When the medium was changed, nonadherent cells were removed, whereas adherent cells were cultured. When culture dishes became nearly confluent, cells were passaged with trypsin-ethylenediaminetetraacetic acid (EDTA).

#### 2.2.3. Flow Cytometry Analysis

hMSSM-derived cells at passage 0 (P0), passage 1 (P1), passage 2 (P2), or passage 3 (P3) were analyzed by flow cytometry for the expression of mesenchymal progenitor cells (MPC) markers following the same procedure described by Berbéri et al. 2016 [24]. Briefly, approximately 10^5^ cells were suspended in 50 *μ*L PBS supplemented with 0.5% human serum albumin (HSA) and 2 mM EDTA, in order to block Fc receptors. Cells were then labeled with antibodies for different cell surface markers: APC-STRO-1, FITC-CD44, PE-CD90, PE-Cy7-CD105, BV-CD73, and PE-CD34 (Biolegend, San Diego, USA) for 30 min at 4°C in the dark. Appropriate fluorochrome-conjugated murine antibodies were used as negative isotype controls. After labeling, cells were washed and suspended in PBS (0.5% HSA, 2 mM EDTA). Samples were acquired using a BD FACSAria (BD biosciences, San Jose, USA) and analysed by Flowjo software (FlowJo, LLC, Oregon, USA).

#### 2.2.4. Osteogenic Differentiation of hMSSM-Derived Cells

At P3, hMSSM-derived cells were examined for their osteogenic potential following the same procedure described by Berbéri et al., 2016 [24]. Briefly, a total of 12-well plates, at 10^5^ cells per well, were cultured for 28 days. The control group was cultured in nonosteogenic normal media (*α*-MEM, 10% FBS, 1% PS), whereas the experimental group was cultured in osteogenic (OS) differentiation media (*α*-MEM, 10−8 dexamethasone, 10−2 *β*-glycerophosphate, and 100 *μ*g/mL L-ascorbic acid 2-phosphate) with media replacement every 2 days.

#### 2.2.5. Osteogenic Differentiation of Scaffold-Embedded hMSSM-Derived Cells

Scaffolds were loaded with 3 × 10^4^ cells (passage 3), placed in 24-well plates, and then cultured in osteogenic (OS) differentiation media (*α*-MEM, 10^−8^ dexamethasone, 10^−2^  *β*-glycerophosphate, and 100 *μ*g/mL L-ascorbic acid 2-phosphate) for 21 days with media replacement every 2 days. Cells cultured alone (without scaffolds) were used as a control.

### 2.3. Biochemical Analyses

#### 2.3.1. MTT Assay

The number of viable cells on the scaffold was determined after 3, 7, 12, 14, 21, 26, and 28 days using the* 3-(4,5-dimethylthiazole-2-yl)-2,5-diphenyltetrazolium bromide *(MTT) assay. The results were expressed as the means of the absorbance data. In brief, cells alone (1 × 10^4^) and cell-seeded scaffolds (1 × 10^4^ cells/scaffold) were placed in culture medium containing 0.5 mg/ml MTT (Sigma-Aldrich, USA) and incubated in a humidified atmosphere at 37°C for 3 h. Viable cells are able to reduce MTT into formazan crystals. The resulting formazan crystals were solubilized in 0.5 ml DMSO (Sigma-Aldrich, USA), and absorbance was recorded at 570 nm using an Enzyme Linked Immunosorbent Acid (ELISA) plate reader.

#### 2.3.2. Alizarin Red Staining Assay

We followed the procedure described in Berbéri et al., 2016 [24]. Briefly, control and experimental groups were evaluated for calcium production at 7, 14, and 21 days of treatment by staining with alizarin red solution, a dye that binds to calcium salts. Indeed, Alizarin red is an anthraquinone derivative used to identify calcium containing osteocytes in a differentiated culture of both human and rodent MSCs. Briefly, cells alone and cell-seeded scaffolds were fixed on ice for 1 hour with 70% ethanol, stained for 30 minutes with 2% alizarin red solution (Sigma AB, Malmö, Sweden), and washed three times with ultrapure water. To quantify the staining, 1 mL of 10% cetylpyridinium chloride (CPC) (Sigma AB, Malmö, Sweden) was added to each well and incubated for 20 min to elute the stain. The eluted stain was read at 550 nm using a spectrophotometer. A standard curve was prepared using alizarin red stain and CPC. The calcium deposition was expressed as molar equivalent of calcium since one mole of alizarin red binds to two moles of calcium in an alizarin red S-calcium complex.

#### 2.3.3. Alkaline Phosphatase (ALP) Activity Assay

We followed the procedure described by Berbéri et al., 2016 [24]. Expression of ALP, a typical osteoblast marker, was measured using an Alkaline Phosphatase Colorimetric Assay Kit (Abcam plc, Cambridge, UK) which uses p-nitrophenyl phosphate (pNPP) as a phosphatase substrate. Intracellular activity of ALP was assessed in control and experimental cultures at 7, 14, and 21 days after treatment. Briefly, cells from both groups were washed with PBS and lysed using ALP assay buffer. Thereafter, a total of 80 *μ*L of the cell lysate was added to 50 *μ*L pNPP in a 96-well plate, and the samples were shielded from direct light for 1 h at room temperature. Following that, 20 *μ*L stop solution (3N NaOH) was added to the wells and the plate was read at 405 nm using an Enzyme Linked Immunosorbent Acid (ELISA) plate reader. Results were expressed as nM p-NP/ml/min and normalized to protein content as measured by the Lowry method in corresponding wells. The Lowry method is used to estimate the amount of protein in a given sample; the total protein concentration is displayed by a color change of the sample solution in proportion to protein concentration.

### 2.4. Quantitative Real-Time-Polymerase Chain Reaction (RT-PCR) Assay

We followed the method described by Berbéri et al., 2016 [24]. hMSSM-derived cells were subjected to real-time PCR in order to examine the mRNA expression of specific osteoblastic markers such as Runx2, osteonectin (ON), osteocalcin (OCN), osteopontin (OPN), bone morphogenetic protein-2 (BMP-2), and type 1 collagen (COL1). Primers used were the following: Runx2 F: CCGCACGACAACCGCACCAT and Runx2 R: CGCTCCGGCCCACAAATCTC; ON, F: CCTGGAGACAAGGTGCTAACAT and R: CGAGTTCTCAGCCTGTGAGA; OCN, F: TCACACTCCTCGCCCTATTGG and R: TCACACTCCTCGCCCTATTGG; OPN, F: AGACCCCAAAAGTAAGGAAGAAG and R: GACAACCGTGGGAAAACAAATAAG; BMP-2, F: GTGTCCCCGCGTGCTTCTTAG and R: ACTCCTCCGTGGGGATAGAAC Col1 F: GAGGGCCAAGACGAAGACATC and Col1 R: CAGATCACGTCATCGCACAAC. Briefly, total RNA was isolated from control and experimental cultures at 7, 14, and 21 days of treatment with Trizol reagent (Invitrogen) according to manufacturer's instructions. First, strand cDNA was synthesized from 1 *μ*g of extracted RNAs using the Revert Aid 1st Strand cDNA synthesis kit (Fermentas). After ss cDNA synthesis, PCR was performed using 1 *μ*g of cDNA mixed with 10 *μ*L Syber green and loaded in duplicates with 5*μ*M forward and reverse primers. PCR cycling conditions were as follows: initial denaturation at 95°C for 10 min, then 45 cycles with denaturation at 95°C for 15s, annealing temperature for 15s, and extension at 72°C for 15s. Basic expression levels for the genes of interest were quantified after normalization to hGAPDH mRNA levels, using human specific primers (h-GAPDH Housekeeping Gene Set) (Roche Applied Science, Branford, USA).

### 2.5. Statistical Analysis

Data are presented as means ± SEM of at least three independent experiments and analysed using Student's* t*-test. P-Values < 0.05 (*∗*) and < 0.01(*∗∗*) were considered significant.

## 3. Results

### 3.1. Culture and Characterization of hMSSM-Derived Cells

**Figure 1:**
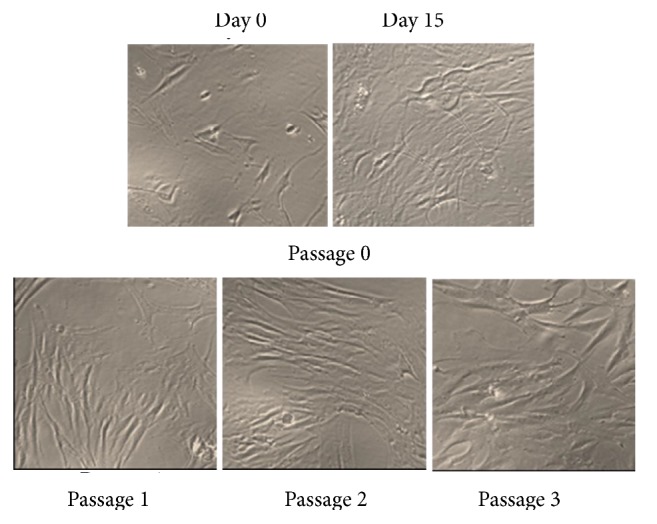
Establishment and characterization of adherent spindle shaped hMSSM cells in culture (10x magnifications).

hMSSM cells were cultured in nonosteogenic culture media. On the next day, nonadherent cells were removed, and all adherent cells showed spindle-shaped fibroblast-like morphology. Around day 15, cells were ~90% confluent and ready to be passaged. Subcultured hMSSM cells replated at 30% confluence in new flasks attached uniformly throughout the culture flasks. Typically, 80–90% confluence was reached by day 8-10 for most of the passaged cells. hMSSM cells were subcultured again until passage 3 (Figure 1). Cells of passages 1 and 2 were retained and stored in liquid nitrogen for further use in this study. It is noteworthy that, in all tested passages (P0, P1, P2, and P3), hMSSM-derived cells were positive for STRO-1, CD44, CD90, CD105, and CD73 but negative for the hematopoietic marker CD34 (data not shown).

### 3.2. Effect of Gelatin, Collagen, and HA/*β*TCP/Fibrin Scaffolds on hMSSM-Derived Cells Viability

To evaluate the viability of hMSSM-derived cells after being seeded on the gelatin, collagen, and HA/*β*TCP/Fibrin scaffolds, an MTT test was performed 7, 14, and 21 days after culturing. This colorimetric assay determines cell viability by measuring mitochondrial activity. Viable cells, containing active NAD(P)H-dependent oxidoreductase enzymes, can reduce yellow MTT to purple formazan crystals. The production of formazan is proportional to the number of viable cells.

**Figure 2:**
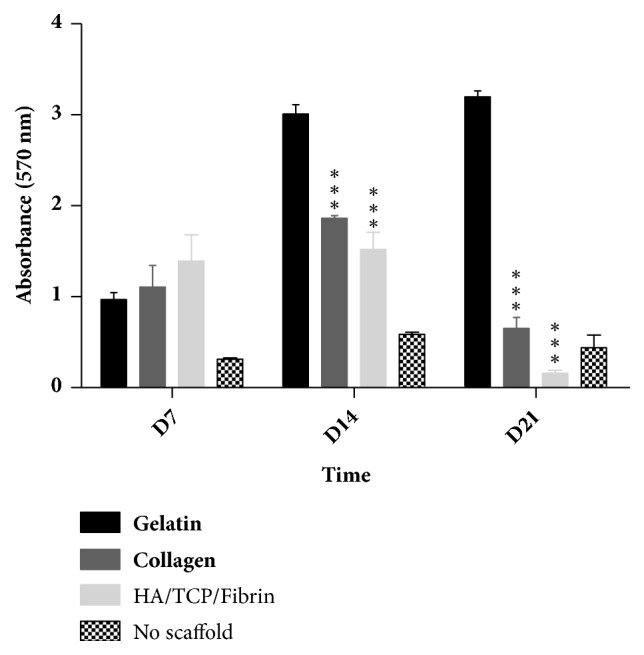
**Impact of scaffold type on hMSSM-derived cells viability.** Cells were seeded either alone or on collagen, gelatin, or HA/*β*TCP/Fibrin scaffold and cultivated for 21 days. MTT assay was used to assess their viability. Each value represents a mean ± SEM for three independent experiments (n=3) each done in triplicate. *∗∗∗p*<0.001* vs.* cells with gelatin scaffold (Student's* t*-test).

At day 7, hMSSM-derived cells seeded on gelatin, collagen, or HA/*β*TCP/Fibrin showed a significantly higher viability than control cells (cultured without scaffold) (Figure 2). At day 14, gelatin had the highest viability, followed by collagen and HA/*β*TCP/Fibrin. After 21 days, the viability of hMSSM-derived cells seeded on collagen or HA/*β*TCP/Fibrin was strikingly reduced. However, cells seeded on collagen maintained robustly high viability (Figure 2). These observations indicate that, among the tested scaffolds, gelatin scaffold was the best to support hMSSM-derived cells viability.

### 3.3. Effect of Gelatin, Collagen, and HA/*β*TCP/Fibrin Scaffolds on hMSSM-Derived Cells-Mediated Calcium Deposition

**Figure 3:**
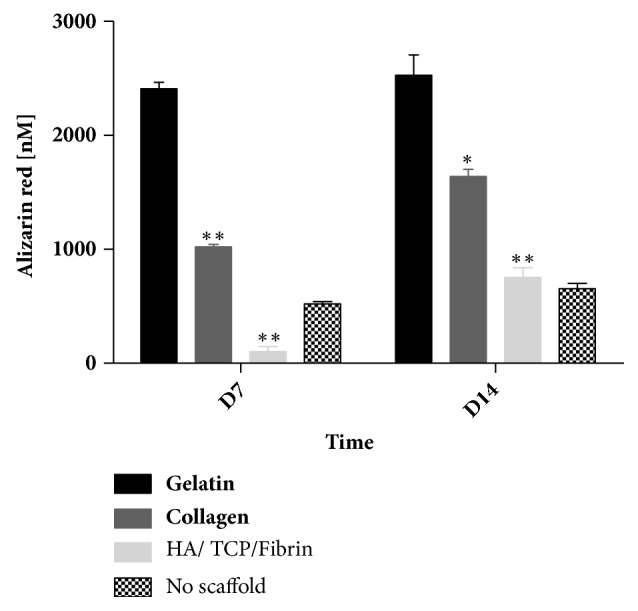
**Impact of scaffold type on calcium deposition by hMSSM-derived cells.** Cells were seeded either alone or on collagen, gelatin, or HA/*β*TCP/Fibrin scaffold and cultivated in osteogenic differentiation medium for 14 days. Alizarin red quantification was used to assess calcium deposition. Each value represents a mean ± SEM for three independent experiments (n=3) each done in triplicate. *∗p*<0.05; *∗∗p*<0.01* vs.* cells with gelatin scaffold (Student's* t*-test).

To evaluate the impact of the different tested scaffolds on hMSSM-derived cells osteogenic differentiation, alizarin red staining assay was performed 7 and 14 days after culturing in osteogenic medium. Alizarin red is used stain to identify calcium in osteocytes arising after differentiation of hMSSM-derived cells. Interestingly, at day 7 as well as at day 14, hMSSM-derived cells, seeded on gelatin scaffold, showed a significantly higher calcification ability than collagen or HA/*β*TCP/Fibrin-scaffold embedded hMSSM-derived cells (Figure 3). This observation indicates that gelatin scaffold was superior in terms of enhancing calcium deposition by hMSSM-derived cells.

### 3.4. Effect of Gelatin, Collagen, and HA/*β*TCP/Fibrin Scaffolds on hMSSM-Mediated Transcription of Runx2, ON, OCN, OPN, BMP-2, and BSP

**Figure 4:**
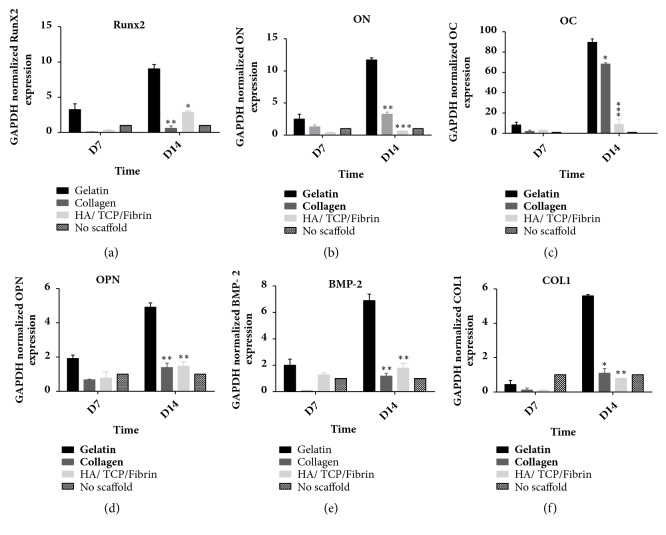
**Effect of scaffold type on hMSSM-derived cells-mediated transcription of different osteoblastic markers. **Cells were seeded either alone or on collagen, gelatin, or HA/*β*TCP/Fibrin scaffold and cultivated in osteogenic differentiation medium for 14 days. Quantitative real-time PCR was used to assess the mRNA levels of Runx2 (Panel (a)), ON (Panel (b)), OCN (Panel (c)), OPN (Panel (d)), BMP-2 (Panel (e)), and COL1 (Panel (f)). Data were normalized to GAPDH levels. Each value represents a mean ± SEM for three independent experiments (n=3) each done in triplicate. *∗p*<0.05; *∗∗p*<0.01, *∗∗∗p*<0.001* vs.* cells with gelatin scaffold (Student's* t*-test).

In a second step, we examined the effect of the different tested scaffolds on cells'-mediated transcription of distinct osteoblastic markers (Runx-2, ON, OCN, OPN, BMP-2, and COL1). Quantitative real-time PCR was performed 7 and 14 days after culturing scaffold-embedded hMSSM-derived cells in osteogenic medium. Remarkably, at each time point, Runx-2, ON, OCN, OPN, BMP-2, and COL1 mRNA levels were higher in gelatin scaffold-embedded hMSSM-derived cells than in hMSSM-derived cells attached to collagen or Ha*β*/TCP scaffolds (Figure 4). These results indicate that gelatin scaffold had the most osteoinductive potential.

### 3.5. Effect of Gelatin, Collagen, and Ha*β*/TCP Scaffolds on Alkaline Phosphatase Activity (ALP) in hMSSM-Derived Cells

**Figure 5:**
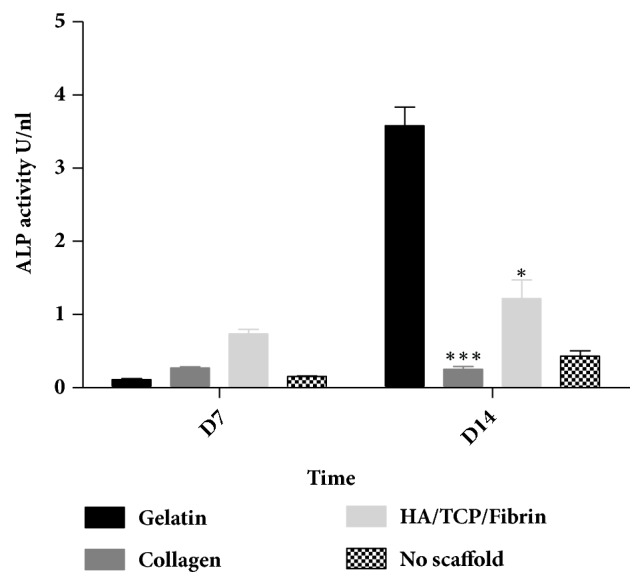
**Impact of scaffold type on ALP activity within hMSSM-derived cells. **Cells were seeded either alone or on collagen, gelatin, or HA/BTCP/FIBIN scaffold and cultivated in osteogenic differentiation medium for 14 days. ALP activity was measured after 7 and 14 days. Each value represents a mean ± SEM for three independent experiments (n=3) each done in triplicate. *∗p*<0.05; *∗∗∗p*<0.001* vs *cells with gelatin scaffold (Student's* t*-test).

We further assessed ALP activity following 7 and 14 days of culturing scaffold-embedded hMSSM-derived cells in osteogenic medium. In parallel with the above results, and after 14 days, ALP activity was more striking in gelatin scaffold-embedded hMSSM-derived cells than in cells seeded on collagen or HA/*β*TCP/Fibrin scaffolds (Figure 5).

## 4. Discussion

Nowadays, significant progress is being made in designing attractive alternatives to autologous bone grafting through the development of* in vitro* biological bone grafts. This is achieved by cultivating osteogenic-progenitor cells within 3D scaffolds, under conditions favoring bone formation [13]. MSCs are present in different fetal and adult tissues including bone marrow (BM), adipose tissue (AT), and periosteum, characterized by high self-renewal capacity and multilineage differentiation potential, and considered as the most common source of osteoprogenitor cells [42]. So far, BM-MSCs and AT-MSCs represent the most commonly studied MSCs for their bone regeneration potential [43]. Lately, a new type of MSCs, being derived from Human Maxillary Schneiderian Membrane (hMSSM), was reported [24, 36]. Interestingly,* in vitro* and* in vivo* studies revealed that hMSSM-derived cells are capable of differentiating to the osteogenic lineage [24, 25, 36, 37]. In this work, we studied, under* in vitro* controlled conditions, the osteogenic potential of hMSSM-derived cells embedded within three different scaffolds (collagen, gelatin, and HA/BTCP/FIBIN). Isolated and cultured hMSSM-derived cells were first validated for their spindle-shaped morphology and expression of MSCs markers. An ideal scaffold should act as an osteoconductive material and support the proliferation and differentiation of stem cells. Here, and despite the fact that the different examined scaffolds were capable of sustaining cell viability during a determined culture period, this capacity was uneven with the gelatin scaffold, ensuring hMSSM-derived cells viability for longer periods than that supported by collagen or HA/*β*TCP/Fibrin scaffolds.

Differentiation towards osteoblasts is a complex process regulated by a number of key components and signaling events. Among the involved factors is runt-related transcription factor 2 (RUNX2) which is considered the master switch of osteogenic differentiation. Runx2 is essential for the formation of a mineralized tissue [44, 45] and its expression status is usually assessed during the early phases of osteogenic differentiation. Another important factor is alkaline phosphatase (ALP), which is crucial for extracellular matrix (ECM) mineralization [46]. Besides ALP mRNA and protein expression levels, the enzymatic activity of ALP is most commonly assayed to determine osteogenic differentiation progression. Moreover, collagen type I (COL1), the main component of the organic part of the ECM, as well as OPN and OCN, two noncollagenous bone ECM proteins, are commonly used as markers of osteogenic differentiation [47]. Furthermore, and differently from undifferentiated MSCs, differentiated osteoblasts accumulate massive extracellular calcium deposits. This osteoblast-mediated mineralization process is indicative of bone formation.

In this study, gelatin scaffold showed higher osteoinductive potential than the other two studied scaffolds. For instance, calcium deposition, a key function of osteoblasts, was more prominent in the case of gelatin scaffold-embedded cells than other cells. Moreover, ALP activity was more striking in cells seeded in gelatin than collagen or HA/*β*TCP/Fibrin scaffold. Further, the transcriptional levels of Runx-2, ON, OCN, OPN, BMP-2, and COL1 were higher in the case of gelatin scaffold-embedded cells than other conditions.

The chemistry and the architecture of the different used scaffolds clearly influenced the potential of osteogenic differentiation. Collagen, as a natural derived scaffold, has been used for bone formation due to its porous, abundant, biodegradable, and biocompatible material [48]. However, several limitations render it as a less desirable scaffold. For instance, despite its ability to support osteoblast differentiation and function,* in vitro*, the poor mechanical property of collagen scaffolds excludes them from being applied in load-bearing sites [48]. Moreover, previous studies showed that the utility of collagen scaffolds,* in vitro*, is challenged by their rapid degradation [48, 49]. Hydroxyapatite *β*-Tricalcium phosphate (Ha*β*-TCP), as a synthetic bone substitutes, is composed of Hydroxyapatite (HA) and Tricalcium phosphate (TCP) at a specific ratio. Fibrin, due to its poor mechanical properties, can be used to coat more stable scaffolds such as Ha*β*-TCP and thus facilitate cell adhesion and distribution over the entire scaffolds. Although Ha*β*-TCP scaffolds are known for their ability to induce osteogenic differentiation, it is well demonstrated that cell viability, proliferation, and differentiation supported by Ha*β*-TCP could vary depending on the Ha*β*/TCP ratio [50].

Hemostatic gelatin sponges have been well described as a suitable* in vitro* model for generating 3D-human and -bovine chondrocyte cultures [51–53]. Moreover, gelatin sponges have been demonstrated to act as a carrier of fibroblast growth factor, and also as an implant for bone regeneration, and thus found to be useful for repairing gingival recession and bone defects [54–56]. Recently, gelatin sponges have been demonstrated for their slow biodegradation (structure stability), biocompatibility, cellular proliferation, cellular migration, and ability to induce osteogenic differentiation of preosteoblasts [57]. This data is consistent with our results indicating that gelatin sponge is a suitable scaffold for osteogenic differentiation and thus bone tissue regeneration.

In fact, the potential application of stem cells in human dentistry is still under investigation. For instance, a previous study comparing early bone formations in patients, with a bilateral highly atrophic posterior maxilla, being grafted with xenogenic sinus graft material (bovine bone material, BBM) alone or BBM admixed with a concentrate of MSCs revealed that MSCs have no positive impact on the new bone formation [58, 59]. On the other hand, there is a growing literature showing that stem cells paired with osteoconductive scaffolding materials can be successfully applied for maxillary sinus lifting as well as bone regeneration [60–66]. Although our obtained results suggest that scaffold-embedded hMSSM-derived cells could support bone regeneration following sinus lift, a major limitation of this study would be that the observed,* in vitro*, osteoinductive potential of the tested scaffold-embedded hMSSM-derived cells does not necessarily translate into* in vivo* applicability, thereby limiting their clinical application.

## 5. Conclusion

In this work, we showed that gelatin scaffold is superior to collagen and HA/*β*TCP/Fibrin scaffolds, in terms of inducing osteogenic differentiation of hMSSM-derived cells. A further* in vivo* study is required to confirm the efficacy of gelatin scaffold-embedded hMSSM-derived cells, in terms of bone regeneration.

## Data Availability

The data used to support the findings of this study are included within the article.
